# Pharmacokinetics and Pharmacodynamics Estimation of Eculizumab in a 2-Year-Old Girl With Atypical Hemolytic Uremic Syndrome: A Case Report With 4-Year Follow-Up

**DOI:** 10.3389/fped.2019.00519

**Published:** 2019-12-17

**Authors:** Ken Saida, Tsuyoshi Fukuda, Kana Mizuno, Masao Ogura, Koichi Kamei, Shuichi Ito

**Affiliations:** ^1^Division of Nephrology and Rheumatology, National Center for Child Health and Development, Tokyo, Japan; ^2^Division of Clinical Pharmacology, Cincinnati Children's Hospital Medical Center, Cincinnati, OH, United States; ^3^Department of Pediatrics, Yokohama City University Graduate School of Medicine, Yokohama, Japan

**Keywords:** eculizumab, atypical hemolytic uremic syndrome, pharmacokinetics, pharmacodynamics, *C3* mutation

## Abstract

**Background:** Eculizumab has dramatically changed poor outcomes of complement-mediated atypical hemolytic uremic syndrome (aHUS) as first-line treatment. Discontinuation of eculizumab remains challenging, and doctor's visits every 2 weeks for intravenous injection because of standard dosing protocols is a huge burden. The Ultra-high cost of eculizumab is also an issue. We attempted to establish a personalized dosing regimen of eculizumab based on pharmacokinetics and pharmacodynamics in a 2-year-old girl with aHUS with a *C3* mutation.

**Case presentation:** She developed aHUS at 5 months of age and was successfully treated with eculizumab. At 2 years of age, we measured eculizumab concentrations and performed pharmacokinetics and pharmacodynamics analysis to optimize her dosing protocol. Her blood concentrations at every 2-, 3-, and 4-week intervals were simulated. Pharmacokinetics analysis showed that her eculizumab clearance was 40% lower than the population mean reported for aHUS. Pharmacokinetic simulation suggested that the 2- and 3-week interval regimen could be sufficient to achieve an efficient trough concentration (>100 μg/mL). We simulated her individual pharmacokinetics profile at 4 years of age with consideration of her growth, which still showed complete inhibition of the alternative complement pathway with the 3-week interval regimen. We continued the 300-mg eculizumab infusion every 3 weeks while CH50 levels were constantly maintained at undetectably low concentrations with no recurrence until 6 years of age.

**Conclusions:** Pharmacokinetics and pharmacodynamics estimation was useful for establishing a personalized dosing regimen for eculizumab and reducing the patient's burden and high medical costs.

## Introduction

Eculizumab (ECZ, Soliris®) has dramatically changed poor outcomes of complement-mediated atypical hemolytic uremic syndrome (aHUS) and is a first-line and promising long-term treatment for aHUS (1, 2). However, there is an increased risk of meningococcal infection, and its ultra-high costs and need for intravenous administration every 2 weeks (usually for patients weighing ≥10 kg), potentially for a lifetime, are a huge burden for patients, especially for children. Therefore, a personalized dosing regimen seems to be a viable solution for reducing the burden.

Frequent blood tests for children are unfavorable, and the measurement of ECZ concentrations is not readily available. Thus, estimation of ECZ concentrations via pharmacokinetics (PK) and pharmacodynamics (PD) analysis is a good option. There are some reports regarding PK/PD analysis in patients with aHUS; however, those data are limited to infants or young children with a low body weight (3–6). Herein, we attempted to establish a personalized dosing regimen of ECZ based on PK/PD in a 2-year-old girl with aHUS and successfully followed up for 4 years in accordance with our prediction.

## Case Presentation and PK/PD Analysis

The patient was a 2-year-old girl who was diagnosed with aHUS with a *C3* mutation (NM_000064.3, c.3313A>C, p.Lys1105Gln) (7). She first developed aHUS at 5 months of age and was successfully treated with ECZ following 3 days of hemodialysis and 3 times of plasma exchange in an acute phase. A blood transfusion was performed once. Maintenance therapy with an ECZ injection every 3 weeks was started subsequently according to the ECZ treatment protocol. At 2 years of age [body weight (BW), 14 kg], measurement of ECZ revealed that trough ECZ concentrations were 103 and 127 μg/mL, and peak concentrations (at the end of ECZ administration) were 386 and 478 μg/mL, respectively. Then, this patient's ECZ PK parameters such as systemic clearance and the volume of distribution were estimated based on a one-compartment model with reported population PK parameter estimates using the Bayesian estimation algorithm as previously described (Supplementary Table 1) (8). We simulated blood concentration profiles of ECZ by administering 300 mg at 2-, 3-, and 4- week intervals. Based on the past reports of target trough concentrations of 50–100 μg/mL (9, 10), ECZ treatment protocols (intervals) were determined to achieve a safe target trough ECZ level of 100 μg/mL.

**Figure 1:**
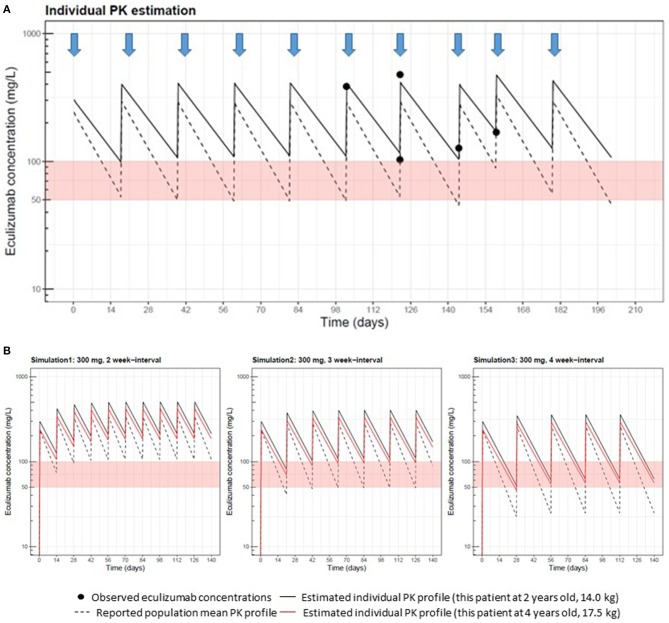
Measurements of eculizumab (ECZ) concentrations and pharmacokinetic (PK) estimation performed at Cincinnati Children's Hospital Medical Center. **(A)** Black circles show ECZ concentrations observed in this patient at 2 years old, receiving 300 mg doses of eculizumab, as indicated by arrows. The solid line represents the individual PK curve estimated with the Bayesian estimation method using MW-Pharm3.82 and five well-captured observations. The dashed line represents the population mean PK curve based on a body weight of 14 kg. As prior PK information in Bayesian estimation, we used reported PK parameters (clearance: 14.6 mL/h per 70 kg; volume of distribution: 6.16 L per 70 kg) for patients with aHUS (U.S. Food and Drug Administration approval document) and inter-individual variability (9). This patient's clearance estimate allometrically adjusted by body weight was 8.7 mL/h per 70 kg, which was lower than the population mean. Estimated volume of distribution (4.9 L per 70 kg) was comparable to the reported population value. **(B)** This patient's eculizumab PK after 300 mg repeated dosing with 2-, 3-, and 4- week interval regimens were simulated using individual PK parameter estimates obtained in **(A)**, shown as black and red solid lines at 2 years of age (14.0 kg) and 4 years of age (17.5 kg), respectively. Population mean PK profiles at 2 years of age are also displayed in dashed lines to compare.

As a result, this patient's estimated PK profile was demonstrated (Figure 1A). The patient had lower ECZ clearance than the population mean (adjusted for weight) reported for aHUS (8.7 mL/h/70 kg vs. 14.6 mL/h/70 kg) (9). PK simulations for this patient at 2 years of age showed that trough concentrations at 2- or 3-week dosing intervals were higher than the target concentrations (≥100 μg/mL) (Figure 1B). This finding indicated that a 2- and 3-week interval regimen could be sufficient for achieving an efficient trough concentration (≥100 μg/mL); however, a 4-week interval regimen was associated with an ~60-μg/mL trough concentration for this patient. On the basis of these simulation results, we continued a 300 mg ECZ infusion at a 3-week interval. The patient was followed up, and laboratory analyses of the levels of red blood cells, hemoglobin, platelets, lactate dehydrogenase, blood urea nitrogen, creatinine and cystatin C, and urinalysis were performed. CH50 was also monitored for reassurance of ECZ effectiveness. No recurrence of aHUS was reported for 2 years with CH50 at undetectably low levels. Low or undetectable CH50 levels (<12%) suggest that the complement system was suppressed, which means the effect of ECZ is sufficient.

In 2 years, PK simulation was performed again at 4 years of age (BW 17.5 kg) with adjustment of individual PK parameters based on body weight increase according to the observed covariate relationship between PK parameters and body weight (9). Reanalysis of PK suggested that the ECZ trough level at the 3-week interval was still >100 μg/mL (Figure 1B, red lines). Therefore, we continued the 3-week infusion of ECZ. Furthermore, at 5 years of age (BW 18 kg), trough concentrations of ECZ at 5 years of age (3-week interval) were measured again and confirmed to be 130 μg/mL and still maintaining >100 μg/mL. Consistent with these clinical observation, CH50 was at consistently undetectable levels. No recurrence was reported since the first occurrence of aHUS at 6 years of age, and the 3-week interval infusion of ECZ is still ongoing.

## Discussion

We applied to establish a personalized dosing regimen of ECZ based on PK/PD analysis for a 2-year-old girl with aHUS and successfully continued maintenance therapy for 4 years. Simulated ECZ concentrations were reliable, as confirmed by measuring ECZ concentrations at 5 years old and performing blood test analyses that included CH50. These results indicate that PK/PD analysis is a very useful option for patients, even for children with aHUS. Considering the difficulty of measuring ECZ concentrations, even only CH50 monitoring may be enough for the follow-up of patients who require ECZ administration in the clinical setting.

The target trough concentrations of ECZ for patients with aHUS were considered to be 50–100 μg/mL (9, 10). Volokhina et al. reported that ≥50 μg/mL is an adequate ECZ concentration for maintenance treatment of patients with aHUS (10). The authors demonstrated ECZ concentrations in 11 patients with aHUS and suggested the possibility of individualized treatment. Five patients were children, including 2- and 1.3-year-old children (body weight, 15 and 11 kg, respectively). Standard dosing instructions indicate that the regimen of ECZ should be 300 mg every 3 weeks for young children weighing <10 kg and 300 mg every 2 weeks for those weighing ≥10 kg. Although, we decided to use the target ECZ trough concentration of 100 μg/mL in this study, the lower levels may reflect adequate complement blockade; therefore, there might be a possibility that a 4-week interval also provides effective complement blockade. To analyze complement inhibition under ECZ treatment, CH50 levels would be a good marker.

Prolonged treatment for patients with aHUS remains a controversial issue, as does ECZ discontinuation since the clinical criteria are not exhaustive (11–14). From the viewpoint of the genetic variant on a molecular basis, the *C3* mutation in our patient (c.3313A>C, p.Lys1105Gln) was located in the C3b thioester-containing domain, which is exactly at the binding site of the complement factor H (CFH) and C3b, potentially disrupting CFH binding to C3 (7). Generally, the long-term outcome is better in *C3* mutations than in *CFH* mutations (15, 16), even though there seems to be differences among *C3* mutation variants (17). Poorer outcomes occur in patients who have a *CFH* mutation. Although, it is difficult to predict the prognosis of this mutation now, we believed this individualized regimen of ECZ (3-week interval) is a good option for patients with aHUS.

In order to make long-term treatment decisions for patients with aHUS, physicians must consider various factors including effectiveness, safety, adverse effect, and medical cost. In our patient, the 3-week interval administration of ECZ has been continued for 4 years after the patient was >10 kg, which is when the treatment interval is recommended to be shortened to 2 weeks from 3 weeks according to the approved standard indication. As a result, this means that the patient could skip 35 hospital visits and save ∼$200 000 in medical costs. In the chronic phase, we have the option to continue ECZ according to standard protocols or discontinue it. However, we would like to suggest the third option as presented in this study by giving due consideration to an individualized treatment regimen by measuring or estimating the dose with PK/PD analysis. This method allows for personalized treatment of patients, and reduces the patients' burden and high medical costs. This case study will facilitate PK/PD-based eculizumab precision dosing in a larger number of patients to minimize their physical and economical burdens.

Optimal treatment for patients with aHUS should be discussed. Our PK/PD based-dosing strategy promises to be safe and useful for estimating individual ECZ concentrations with sparse sampling data and establishing maintenance treatment for aHUS.

## Data Availability Statement

All relevant data is contained within the article.

## Ethics Statement

The studies involving human participants were reviewed and approved by The Institutional Review Board of National Center for Child Health and Development. Written informed consent to participate in this study was provided by the participants' legal guardian/next of kin.

## Author Contributions

KS drafts the manuscript. TF and KM performed pharmacokinetics and pharmacodynamics analysis of eculizumab made substantial contributions to the conception and design. MO and KK treated the patient while she was hospitalized, and involved in revising the manuscript. SI oversaw the project. All authors approved the final case report as submitted and agree to be accountable for all aspects of the work.

### Conflict of Interest

The authors declare that the research was conducted in the absence of any commercial or financial relationships that could be construed as a potential conflict of interest.
